# Medicaid Coverage of Dental Services and Dental Hygiene During Pregnancy

**DOI:** 10.1001/jamanetworkopen.2025.44148

**Published:** 2025-11-14

**Authors:** Madeline F. Perry, Lynn M. Yee, Joe Feinglass

**Affiliations:** 1 Department of Obstetrics and Gynecology, Northwestern University Feinberg School of Medicine, Chicago, Illinois; 2 National Clinician Scholars Program, University of Pennsylvania, Philadelphia; 3 Division of General Internal Medicine, Northwestern University Feinberg School of Medicine, Chicago, Illinois

## Abstract

This cross-sectional study evaluates the association of level of state Medicaid coverage of dental services with receipt of teeth cleaning during pregnancy.

## Introduction

Periodontal disease has been associated with adverse maternal and neonatal outcomes.^1^ Medicaid coverage for dental services is not mandatory during pregnancy and differs on a state-by-state basis. While Medicaid coverage of dental services is associated with use of dental services during pregnancy, this association has not been evaluated since Patient Protection and Affordable Care Act (ACA) implementation, when dental coverage was expanded.^2,3^ Using post-ACA data, we evaluated the association between the generosity of state Medicaid coverage of dental services and receipt of teeth cleaning during pregnancy in a Medicaid-insured population.

## Methods

This retrospective cross-sectional study used 2016-2020 data from the Pregnancy Risk Assessment Monitoring System (PRAMS) including 44 states linked with 2019-2020 data on dental coverage from the Medicaid and Children's Health Insurance Program Payment and Access Commission. PRAMS are deidentified, publicly available data; thus, this study was deemed exempt by the Northwestern University Feinberg School of Medicine institutional review board. Results reporting adhered to the STROBE reporting guideline. The study included respondents insured by Medicaid at the time of delivery. The exposure was state-level generosity of Medicaid coverage of dental services.^4^ Each state’s Medicaid program was characterized as having no dental coverage, emergency dental coverage only, coverage of 1 to 4 dental services, or coverage of 5 or more dental services (eMethods in Supplement 1).^4^ The outcome was receipt of teeth cleaning during pregnancy. Using multivariable Poisson regression,^5^ we assessed the association between states’ Medicaid coverage of dental services and the likelihood of teeth cleaning during pregnancy, controlling for respondent sociodemographic characteristics.

A sensitivity analysis was performed using the same exposure and covariates for commercially insured respondents (eMethods in Supplement 1). All analyses were based on population-weighted data using the complex survey module of Stata, version 18.

## Results

The analysis included 68 385 respondents representing a weighted population of 3 247 222. Among 44 included state Medicaid programs, 3 (6.8%) provided no dental coverage; 6 (13.6%), only emergency coverage; 6 (13.6%), coverage of 1 to 4 dental services; and 29 (65.9%), coverage for 5 or more dental services. Teeth cleaning during pregnancy was reported by 31.6% of respondents insured by Medicaid. The weighted percentages of respondents reporting teeth cleaning during pregnancy were 19.5%, 23.4%, 23.0%, and 34.4% in states with no Medicaid coverage of dental services, emergency coverage only, coverage of 1 to 4 services, and coverage of 5 or more services, respectively (Table 1). Sociodemographic characteristics are summarized in Table 1. Among Medicaid respondents, in states with no Medicaid coverage of dental services, there was a lower likelihood of teeth cleaning during pregnancy compared with states with Medicaid coverage of 5 or more dental services (the most generous coverage) (adjusted incidence rate ratio [aIRR], 0.59; 95% CI, 0.52-0.66) (Table 2). People living in states with emergency dental services only and those in states with 1 to 4 dental services covered had a lower likelihood of teeth cleaning during pregnancy compared with those with the most generous Medicaid dental coverage (emergency: aIRR, 0.70 [95% CI, 0.65-0.75]; 1-4: aIRR, 0.69 [95% CI, 0.65-0.74]) (Table 2). In sensitivity analysis including respondents who were commercially insured, 58.2% reported receipt of teeth cleaning during pregnancy. State differences among privately insured respondents did not reflect state differences observed among Medicaid respondents.

**Table 1.** Sociodemographic Characteristics of Respondents Insured by Medicaid and Privately Insured by State Medicaid Dental Coverage^a^

Characteristic	Respondents, No. (%)^b^							
	Delivery covered by Medicaid (n = 68 385)^c^				Delivery covered by private insurance (n = 85 571)			
	No dental coverage	Emergency dental only	1-4 Dental services	≥5 Dental services	No dental coverage	Emergency dental only	1-4 Dental services	≥5 Dental services
Living in Medicaid coverage category	3299 (4.7)	7081 (5.8)	8471 (12.8)	49 534 (76.7)	3623 (3.2)	10 267 (6.6)	8434 (8.6)	63 247 (81.6)
Received teeth cleaning	687 (19.5)	1690 (23.4)	2116 (23.0)	17 613 (34.4)	2022 (55.0)	5853 (57.4)	4853 (53.7)	36 414 (58.8)
Age, y								
<20	289 (10.2)	694 (9.6)	756 (8.5)	3697 (7.2)	32 (1.0)	153 (1.3)	86 (1.0)	604 (0.9)
20-29	1888 (62.8)	4277 (62.4)	5202 (62.8)	28 356 (59.0)	1357 (41.7)	4451 (46.5)	3452 (38.8)	20 908 (34.0)
30-39	1048 (25.5)	1987 (26.5)	2350 (26.8)	16 175 (31.4)	2093 (53.5)	5277 (49.1)	4617 (56.4)	38 740 (60.7)
>39	74 (1.6)	123 (1.6)	163 (1.9)	1306 (2.4)	141 (3.8)	386 (3.2)	141 (3.8)	1481 (4.4)
Maternal education								
≤12 y	790 (23.6)	1772 (25.2)	1532 (19.4)	10 334 (19.7)	44 (1.4)	349 (2.6)	141 (1.8)	1481 (2.1)
Completion of high school or GED	1391 (43.7)	2758 (39.2)	3631 (44.7)	3631 (30.3)	412 (13.3)	1622 (13.4)	1034 (12.9)	6212 (10.9)
>12 y	1118 (32.7)	2551 (35.6)	3308 (35.9)	20 318 (40.1)	3167 (85.3)	8296 (84.0)	7259 (85.3)	55 554 (84.0)
Annual income								
≤$20 000	1160 (52.4)	3573 (49.1)	4875 (55.1)	23 780 (46.0)	103 (4.0)	494 (4.4)	354 (4.9)	2911 (4.3)
$20 001-$40 000	783 (23.2)	1914 (29.2)	2108 (24.3)	13 567 (28.7)	388 (12.1)	1408 (13.4)	1043 (11.6)	6248 (9.0)
$40 001-$60 000	197 (6.0)	473 (6.9)	497 (6.3)	3899 (8.8)	531 (16.3)	1984 (20.1)	1409 (16.5)	8401 (12.8)
$60 001-$85 000	53 (1.7)	148 (2.1)	118 (1.6)	1053 (2.4)	690 (16.2)	2117 (20.6)	1731 (19.4)	10 632 (16.3)
>$85 000	1106 (16.7)	973 (12.8)	873 (12.7)	7235 (14.0)	1911 (48.4)	4264 (41.5)	3897 (47.6)	35 055 (57.7)
Maternal race^d^								
American Indian or Alaska Native	10 (0.3)	261 (5.6)	70 (0.06)	2374 (1.7)	5 (<0.1)	277 (1.4)	62 (0.2)	1003 (0.2)
Asian	45 (0.7)	261 (3.0)	70 (0.9)	2374 (4.2)	212 (2.6)	732 (4.9)	212 (2.7)	7527 (7.8)
Black	1264 (36.8)	775 (6.90)	3404 (37.7)	14 903 (25.3)	547 (14.2)	318 (1.5)	796 (10.3)	7222 (8.3)
Native Hawaiian or Other Pacific Islander	1 (<0.1)	17 (0.1)	1 (<0.1)	27 (<0.1)	1 (<0.1)	17 (<0.1)	1 (<0.1)	12 (<0.1)
White	1599 (53.8)	4007 (70.8)	4180 (49.8)	20 537 (57.3)	2720 (80.0)	7711 (84.4)	7085 (81.4)	42 627 (78.9)
Multiracial	187 (3.0)	1033 (8.9)	200 (2.4)	3393 (3.7)	79 (1.8)	961 (5.6)	132 (1.7)	2998 (2.1)
Other^e^	193 (5.2)	460 (4.7)	332 (8.6)	3706 (7.8)	59 (1.2)	251 (2.0)	146 (3.6)	1858 (2.7)
Maternal ethnicity^d^								
Hispanic	584 (13.3)	1617 (24.3)	725 (18.7)	12 229 (22.8)	161 (3.4)	967 (9.2)	440 (12.7)	6536 (8.5)
Non-Hispanic	2715 (86.7)	5464 (75.7)	7746 (81.4)	37 305 (77.2)	3462 (96.6)	9300 (90.8)	7994 (87.3)	56 711 (91.5)
Married	857 (26.5)	2517 (36.1)	2164 (26.5)	16 522 (34.9)	3057 (85.7)	8809 (88.3)	7181 (84.1)	53 610 (84.8)
Uninsured before conception	747 (27.4)	1885 (28.9)	2591 (31.9)	9362 (21.7)	50 (1.2)	189 (1.8)	169 (2.6)	1194 (1.8)

Abbreviations: GED, General Educational Development; PRAMS, Pregnancy Risk Assessment Monitoring System.

^a^ District of Columbia, California, Idaho, Nevada, Ohio, South Carolina, and Texas were excluded from analysis as nonparticipants in PRAMS; the 153 956 respondents represent a weighted estimate of 7 957 338 respondents. No dental coverage: Alabama, Delaware, and Tennessee; emergency coverage only: Arizona, Hawaii, New Hampshire, Oklahoma, Utah, and West Virginia; coverage of 1 to 4 services: Florida, Kansas, Louisiana, Maine, Mississippi, and Wyoming; coverage of 5 or more services: Alaska, Arkansas, Colorado, Connecticut, Georgia, Iowa, Illinois, Indiana, Kentucky, Massachusetts, Maryland, Michigan, Minnesota, Missouri, Montana, North Carolina, North Dakota, Nebraska, New Jersey, New Mexico, New York, Oregon, Pennsylvania, Rhode Island, South Dakota, Virginia, Vermont, Washington, and Wisconsin.

^b^ Estimates are unweighted sample sizes and survey-weighted percentages.

^c^ Dental services that may or may not be covered by Medicaid include diagnostic, preventive, restorative, periodontal, dentures, oral surgery, and orthodontia.

^d^ Race and ethnicity, which are acknowledged to be social constructs, were included as a proxy to assess influence of structural racism. Race and ethnicity data in PRAMS are derived from linked National Vital Statistics System birth certificate data which are self-reported (eMethods in Supplement 1).

^e^ Further categorization is not available in PRAMS.

**Table 2.** Association of Medicaid Coverage of Dental Services With Receipt of Teeth Cleaning During Pregnancy

Insurance coverage during pregnancy	Adjusted incidence rate ratio (95% CI)^a^			
	No dental coverage	Emergency coverage only	1-4 Dental services	≥5 Dental services
Medicaid	0.59 (0.52-0.66)	0.70 (0.65-0.75)	0.69 (0.65-0.74)	1 [Reference]
Commercial insurance^b^	0.96 (0.91-1.00)	1.02 (0.99-1.04)	0.94 (0.90-0.97)	1 [Reference]

^a^ Adjusted for education, income, age, race and ethnicity, marital status, and no insurance before conception.

^b^ Included 85 571 participants (total weighted population: 4 710 116).

## Discussion

In this study of the post-ACA implementation era, minimal or no Medicaid coverage of dental services was significantly associated with a lower likelihood of receiving teeth cleaning during pregnancy in a Medicaid-insured population. This association did not exist in a commercially insured population, suggesting that differential rates of teeth cleaning were associated with state Medicaid dental policy rather than other state-level characteristics. A limitation is that we did not use methods to determine causal inference and therefore cannot comment on causation. States currently have the opportunity to include dental services as an essential health benefit, which could be a lever to increasing access to dental services during pregnancy.^6^

## Notes

Supplement 1.
eMethods.

Supplement 2.
Data Sharing Statement

## References

[1] Karimi N, Samiee N, Moradi Y. The association between periodontal disease and risk of adverse maternal or neonatal outcomes: A systematic review and meta-analysis of analytical observational studies. Health Sci Rep. 2023;6(10):e1630. doi:10.1002/hsr2.1630 37867783 PMC10587389

[2] Elani HW, Sommers BD, Kawachi I. Changes in coverage and access to dental care five years after ACA Medicaid expansion. Health Aff (Millwood). 2020;39(11):1900-1908. doi:10.1377/hlthaff.2020.00386 33136492 PMC8056359

[3] Lee H, Marsteller JA, Wenzel J. Dental care utilization during pregnancy by Medicaid dental coverage in 26 states: Pregnancy risk assessment monitoring system 2014-2015. J Public Health Dent. 2022;82(1):61-71. doi:10.1111/jphd.12483 34904236

[4] Compendium: state Medicaid fee-for-service adult dental services coverage policies. Medicaid and CHIP Payment and Access Commission. January 2021. Accessed September 1, 2025. https://www.macpac.gov/publication/compendium-states-medicaid-fee-for-service-adult-dental-services-coverage-policies/

[5] Zou G. A modified poisson regression approach to prospective studies with binary data. Am J Epidemiol. 2004;159(7):702-706. doi:10.1093/aje/kwh090 15033648

[6] HHS finalizes policies to make Marketplace coverage more accessible and expand essential health benefits. CMS.gov. April 2, 2024. Accessed September 1, 2025. https://www.cms.gov/newsroom/press-releases/hhs-finalizes-policies-make-marketplace-coverage-more-accessible-and-expand-essential-health

