# Supplementary material for: Medicaid Coverage of Dental Services and Dental Hygiene During Pregnancy
Source: JAMA Netw Open. 2025 Nov 14;8(11):e2544148. doi: 10.1001/jamanetworkopen.2025.44148 (PMC12619092; doi:10.1001/jamanetworkopen.2025.44148)
Supplement: Supplement 1. — eMethods. [file jamanetwopen-e2544148-s001.pdf]

Supplemental Online Content

Perry M, Yee LM, Feinglass J. Medicaid coverage of dental services and dental hygiene during pregnancy. *JAMA Netw Open*. 2025;8(11):e2544148. doi:10.1001/jamanetworkopen.2025.44148

eMethods.

This supplemental material has been provided by the authors to give readers additional information about their work.

© 2025 Perry et al. *JAMA Network Open*.

eMethods

Defining exposure

Medicaid coverage of dental services was determined through Medicaid and CHIP Payment and Access Commission (MACPAC) data from 2019-2020. MACPAC does not list each dental procedure covered by Medicaid and instead reviews the coverage that is provided for types of services. Types of dental services that may be covered by Medicaid included diagnostic, preventive, restorative, periodontal, dentures, oral surgery, and orthodontia.[1] Our classification schema for generosity of coverage of dental services is based off of MACPAC. MACPAC groups dental coverage as 1) no coverage or emergency only, 2) coverage of 1-4 types of services and 3) coverage of 5 or more services. This is similar to Center for Health Care Strategies (CHCS) categorization of coverage of dental services, defined below[2]:

- Emergency only: Relief of pain under defined emergency situations
- Limited coverage: Fewer than 100 diagnostic, preventive, and minor restorative procedures recognized by the American Dental Association (ADA); per-person annual expenditure for care is $1,000 or less.
- Extensive coverage: A comprehensive mix of services, including more than 100 diagnostic, preventive, and minor and major restorative procedures approved by the ADA; per-person annual expenditure cap is at least $1,000.

As MACPAC does not describe each dental procedure covered, we define increasing generosity by increasing types of services covered and opted to separate no coverage and emergency-only coverage.

If a state’s Medicaid program provided different coverage by Medicaid eligibility group, the more comprehensive coverage was used for categorization, which would bias our results towards the null. A pregnant person’s Medicaid eligibility is not just determined by pregnancy status, and many pregnant people qualify for Medicaid by income, irrespective of pregnancy status.

Defining outcome

Receipt of dental cleaning during pregnancy was a self-reported outcome. Respondents were categorized as having received teeth cleaning during pregnancy if they answered “yes” to the following question: “During your most recent pregnancy, did you have your teeth cleaned by a dentist or dental hygienist?”

Sensitivity analysis

© 2025 Perry et al. *JAMA Network Open*.

We did not expect state Medicaid generosity for dental services to be associated with receipt of dental services among commercially insured people. Thus, sensitivity analysis was performed using the same exposure and individual covariates for commercially insured respondents.

Race and ethnicity

Race and ethnicity were included in this analysis as a proxy to assess the influence of structural racism and systemic disparities on results. The authors acknowledge that race is a social construct. The Pregnancy Risk Assessment Monitoring System (PRAMS) utilizes race and ethnicity that are self-reported on birth certificates. PRAMS provides the following race categories: other Asian, White, Black, American Indian, Chinese, Japanese, Filipino, Hawaiian, other Non-White, Alaskan Native, Mixed Race. Race categories were further specified by the investigators to the following schema: American Indian or Alaskan Native, Asian (including other Asian, Chinese, Japanese, and Filipino), Black, multiple races, Native Hawaiian, other non-White, and White. The "multiple races" category could not be further deconstructed as PRAMS does not include the multiple races that people may self-identify as. The American Indian or Alaskan Native group and Asian group were collapsed as previously defined because of small sample sizes.

© 2025 Perry et al. *JAMA Network Open*.

References

1. Compendium: State Medicaid Fee-for-Service Adult Dental Services Coverage Policies. (https://www.macpac.gov/publication/compendium-states-medicaid-fee-for-service-adult-dental-services-coverage-policies/).
2. Medicaid Adult Dental Benefits: An Overview. (https://www.chcs.org/media/Adult-Oral-Health-Fact-Sheet_091519.pdf).

© 2025 Perry et al. *JAMA Network Open*.
