# Supplementary material for: Medicaid Coverage of Dental Services and Dental Hygiene During Pregnancy
Source: JAMA Netw Open. 2025 Nov 14;8(11):e2544148. doi: 10.1001/jamanetworkopen.2025.44148 (PMC12619092; doi:10.1001/jamanetworkopen.2025.44148)
Supplement: Supplement 2. — Data Sharing Statement [file jamanetwopen-e2544148-s002.pdf]

Data Sharing Statement

Perry. Medicaid Coverage of Dental Services and Dental Hygiene During Pregnancy. *JAMA Netw Open*. Published November 14, 2025. doi:10.1001/jamanetworkopen.2025.44148

Data
Data available: No

Additional Information
Explanation for why data not available: PRAMS data and codebook are publicly available through the CDC. Coding for variable definition and statistical analysis can be provided upon request.
